# Impact of ankylosing spondylitis on stroke limited to specific subtypes: Evidence from Mendelian randomization study

**DOI:** 10.3389/fimmu.2022.1095622

**Published:** 2023-01-19

**Authors:** Jian Mei, Penghui Wei, Linjie Zhang, Haiqi Ding, Wenming Zhang, Yusen Tang, Xinyu Fang

**Affiliations:** ^1^ Department of Orthopaedic Surgery, the First Affiliated Hospital, Fujian Medical University, Fuzhou, China; ^2^ Department of Orthopaedic Surgery, National Regional Medical Center, Binhai Campus of the First Affiliated Hospital, Fujian Medical University, Fuzhou, China; ^3^ Department of Orthopedic Surgery, Experimental Orthopedics, Centre for Medical Biotechnology (ZMB), University of Regensburg, Regensburg, Germany; ^4^ Department of Neurosurgery, Neurosurgery Research Institute, The First Affiliated Hospital of Fujian Medical University, Fuzhou, Fujian, China; ^5^ Institute for Stroke and Dementia Research, University Hospital, Ludwig Maximilian University, Munich, Germany; ^6^ Department of Orthopaedics, The 909th Hospital, School of Medicine, Xiamen University, Zhangzhou, Fujian, China

**Keywords:** Mendelian randomization (MR) analaysis, ankylosing spondylitis (AS), stroke, single nucleotide polymorphisms (SNP), GWAS

## Abstract

**Background:**

The relationship between Ankylosing Spondylitis (AS) and the risk of stroke is complex. Therefore, we utilized Two-Sample Mendelian randomization to examine the probable causal link between these two features.

**Methods:**

The genetic instruments linked to AS were chosen from a summary-level genetic data set from the FinnGen consortium in people of European ancestry (1462 cases and 164,682 controls). Stroke and its subtypes were selected as outcomes, and the MEGASTROKE consortium population was used to identify the genetic associations of AS on stroke (40,585 cases and 406,111 controls), ischemic stroke (IS) (34,217 cases and 406,111 controls), and its subtypes including large artery stroke (LAS) (4373 cases and 146,392 controls), small vessel stroke (SVS) (5386 cases and 192,662 controls), and cardioembolic stroke (CES) (7193 cases and 204,570 controls). Intracerebral hemorrhage (ICH) (1687 cases and 201,146 controls) data set from the FinnGen consortium was also used. To obtain the casual estimates, the inverse variant weighted (IVW) method was mainly used. By examining the heterogeneity and pleiotropy of particular single nucleotide polymorphisms (SNPs), the robustness of the results was also examined.

**Results:**

There was no evidence found to prove the correlation between genetically predicted AS and stroke (odds ratio [OR] 1.014; 95% confidence interval [CI] 0.999-1.031; P = 0.063), ICH (OR 1.030; 95% CI 0.995-1.067; P = 0.090), and IS (OR 1.013; 95% CI 0. 998-1.030; P = 0.090). In terms of the different subtypes of IS, there was strong evidence of positive causal inferences on CES (OR 1.051; 95% CI 1.022-1.081; P = 0.001), and suggestive evidence of positive causal inferences on LAS (OR 1.042; 95% CI 1.003-1.082; P = 0.033), while it was not significant for SVS (OR 1.010; 95% CI 0.975-1.047; P = 0.563).

**Conclusion:**

This study suggests that the possible causative impact of genetically predicted AS on stroke may be restricted to the CES and LAS subtypes.

## Introduction

Ankylosing spondylitis (AS), an autoimmune disorder marked by systemic inflammation, primarily affects the axial skeleton (1). In prior research, AS was revealed to be linked to an elevated risk of ischemic heart disease (2). However, the risk of stroke in patients with AS remains unknown. Reports on the effect of AS on stroke are diverse. Several studies indicate that AS is an independent predictor of stroke (3, 4), while some published contentious results (5, 6). According to the findings of a meta-analysis on the subject of the risk of stroke in arthritis, the risk of stroke in patients with AS is 1.49 times higher than in controls (7) However, Current observational studies are frequently constrained by the potential for confounding and reverse causality. In addition, establishing the potential causative links between AS and stroke is crucial for enhancing stroke prevention.

Mendelian randomization (MR) is a strategy for investigating causal relationships that can efficiently circumvent the aforementioned restrictions by employing genetic variants as exposure (i.e., AS) instrumental variables (IVs) (8). Recently, an MR study explored the causality between AS and atrial fibrillation (AF) (9). Similarly, we investigated the causal inference of genetically predicted AS on stroke, intracerebral hemorrhage (ICH), and ischemic stroke (IS) and its subtypes using summary data from available genome-wide association studies (GWAS).

## Methods

### Study design and data sources

**Figure 1:**
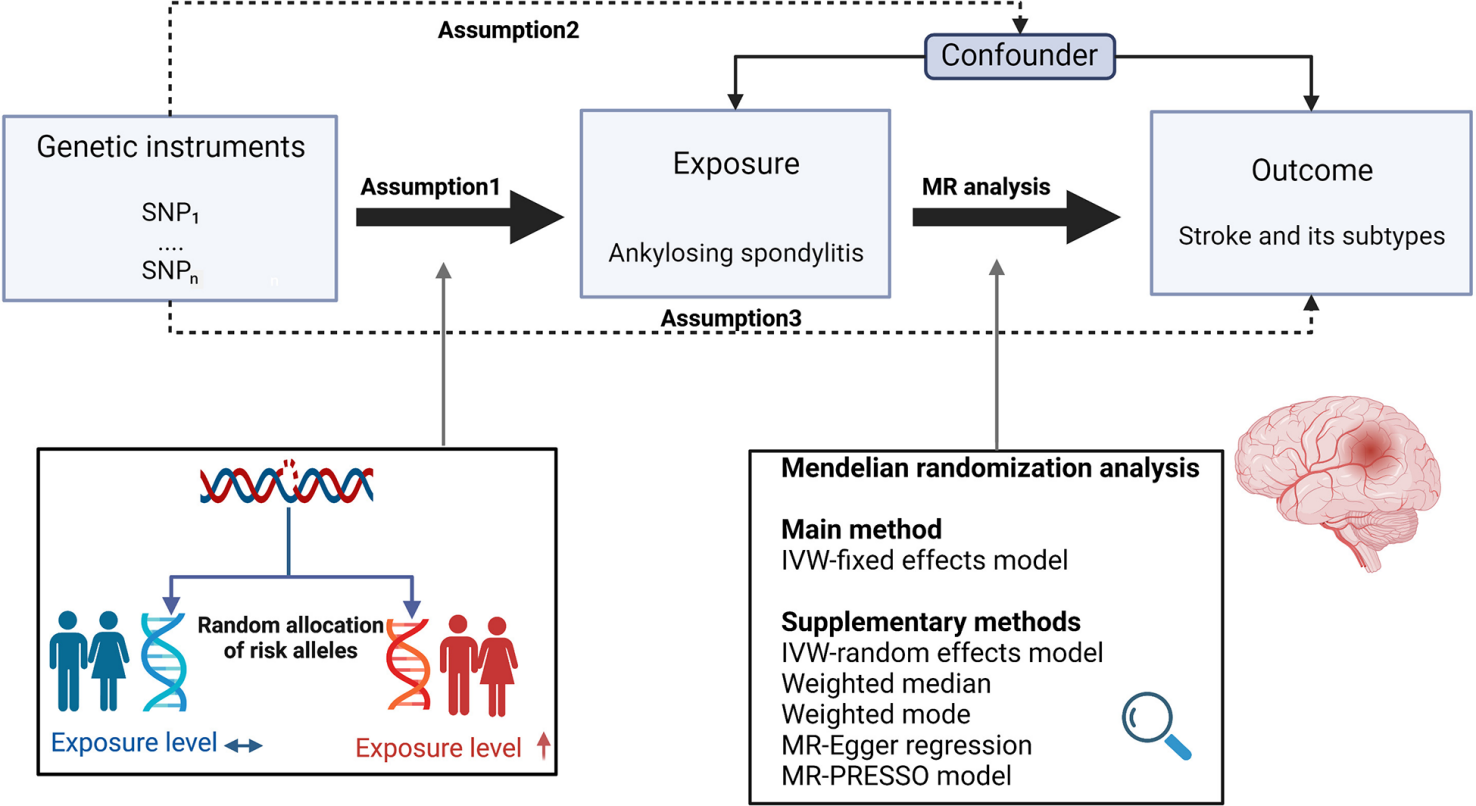
Study design overview and assumptions of the MR design. Assumption 1 indicates that the genetic variants proposed as instrumental variables should be robustly associated with the risk factor of interest, assumption 2 indicates that the used genetic variants should not be associated with potential confounders, and assumption 3 indicates that the selected genetic variants should affect the risk of the outcome merely through the risk factor, not *via* alternative pathways. The MR design can reduce residual confounding and reverse causality, thereby reinforcing the causal inference of an exposure outcome association. The basis of this is that genetic variants, selected as an instrumental variable for studying the effect of modifying the exposure, are randomly allocated at conception and are therefore less vulnerable to confounding from environmental factors and reverse causation. IVW, inverse-variance weighted. (Created with BioRender.com).

**Figure 1** depicts the study design overview and the MR study’s assumptions. Genetic instruments for the exposures (i.e., AS) were obtained from the FinnGen consortium. Study outcomes included stroke, ICH, IS and its subtypes, which were defined based on the Trial of Org 10172 in acute stroke treatment categorization (10), including large artery stroke (LAS), small vessel stroke (SVS), and cardioembolic stroke (CES), whose datasets were from the European-ancestry MEGASTROKE collaboration (11).

**Table 1** provides specifics regarding the data sources used and the demographic profiles of AS, stroke, and its subtypes. As this research was based on previously published GWAS summary data, institutional review board approval was not needed, and prior informed consent was obtained from all participants.

**Table 1:** Data sources and demographic profiles.

Exposures or outcome	Sample size (cases/controls)	Ancestry	Consortia	PubMed ID or URL
**Ankylosing spondylitis**	1,462/164,682	European	FinnGen consortium	(https://www.finngen.fi/fi)
**Stroke**	40,585/406,111	European	MEGASTROKE Consortium	29531354
**ICH**	1687/201146	European	FinnGen consortium	(https://www.finngen.fi/fi)
**Ischemic stroke**	34217/406,111	European	MEGASTROKE Consortium	29531354
**Cardioembolic stroke**	7193/204,570	European	MEGASTROKE Consortium	29531354
**Large artery stroke**	4373/146,392	European	MEGASTROKE Consortium	29531354
**Small vessel stroke**	5386/192,662	European	MEGASTROKE Consortium	29531354

### Genetic instrument selection

To identify genetic IVs that comply with these three MR assumptions, a set of quality control procedures was conducted. Firstly, the Genome-wide significance criterion (*P*<5E–8) was used to select genetic instruments for AS in association studies. Secondly, to reduce the effect of significant linkage disequilibrium (LD), we conducted the clumping procedure with R2 < 0.001 and a window size = 10,000 kb base on the data from the 1000 genomes project in European ancestry (12). Within single nucleotide polymorphism (SNP) pairs with an LD R^2^ larger than the specified threshold, the one with a lower *P* value was retained. Thirdly, SNPs with minor allele frequencies (MAF) of less than 0.01 were also removed. Fourthly, when the targeted SNPs were not located in the outcome GWAS, proxy SNPs that shared high levels of linkage disequilibrium (R^2^ > 0.8) with the target SNPs were searched for instead. Finally, to generate a summary set with each SNP on the exposure and the outcome corresponding to the same effect alleles, we excluded SNPs with non-concordant alleles and palindromic SNPs by harmonizing the exposure and outcome datasets. In addition, to determine the relationship between the selected SNPs and any potential confounders in the potential effect of AS on stroke, for each index SNP, we also searched the PhenoScanner_V2_ website (http://www.PhenoScanner.medschl.cam.ac.uk/) for its potential association with multiple traits using LD traits (set: *P* for trait-associated SNPs < 5E-8, r^2^ for LD > 0.8 in EUR), and those associated with potential confounders were removed. These meticulously selected SNPs served as the final genetic IVs for the following MR analysis.

Furthermore, we used the following equation to obtain the F statistics for each SNP: F=R^2^(N - 2)/(1 - R^2^). R^2^ represents the variance of each collected IV on AS. N is the sample size of the original GWAS research (i.e., AS) (13). To calculate R^2^ for each IV, the following formula was employed: R^2^ = 2β^2^EAF(1−EAF)/2β^2^EAF(1−EAF) + (se(β))^2^ 2NEAF(1−EAF) where EAF represents the effect allele frequency, beta represents the estimated genetic effect on AS, N represents the sample size of the GWAS, and se represents the standard error of the genetic effect (14). IVs with F statistics under ten were regarded as unreliable instruments and were excluded from MR analysis (13).

### MR analysis

For the main MR analysis, fixed effects inverse variance weighted (IVW) methods were used. To assess the reliability of the findings, MR-Egger regression (15) and median-based estimator (weighted median and weighted mode) (16) were also conducted.

### Heterogeneity, pleiotropy, and sensitivity analysis

To assess the likelihood of horizontal pleiotropy, we performed the MR-Egger regression. The intercept term of the MR-Egger regression shows the average pleiotropic effect of the Ivs (15). MR-PRESSO global test was also used to test the robustness of the MR-Egger regression pleiotropy detection result (17). The asymmetry of the funnel plot can also be considered an indicator of horizontal pleiotropy (18). To identify heterogeneity, MR-Egger and IVW in Cochran’s Q statistic were utilized, and Cochrane’s Q value was estimated to assess the heterogeneity among used IVs. Also, a leave-one-out (LOO) analysis was performed to assess whether a single SNP drives the association, and random-effects modes IVW were used, as they provide a more accurate estimation if there is any heterogeneity (18). To determine the reliability of the results, we calculated the statistical power using the mRnd website (https://shiny.cnsgenomics.com/mRnd/). To determine the direction of a possible causal effect between two phenotypes, we further investigated the effect of stroke and its subtypes on AS to check whether our result was affected by any possible reverse causality, the details of the selection of IVs in the stroke-associated GWAS are described in the **Supplementary Methods**.

All statistical analyses were performed using the Two-Sample MR package in R statistical software version 4.2.1. (R Foundation). To account for multiple testing, we considered associations with P values below 0.008 (where P = 0.05/6) to represent strong evidence of causal associations, and associations with P values below 0.05 but above 0.008 were considered to be suggestive evidence of associations in the MR analysis.

## Results

### Selection of IVs

Based on the summary-level data, 13 SNPs were identified as genome-wide and significantly linked with AS. However, in the analysis of the effect of AS on stroke, IS, LAS, SVS, and CES, 3 SNPs were eliminated owing to data not being available in the summary statistic of outcome and there being no suitable proxy SNP to replace(rs181316459), namely palindromic(rs16894011) and incompatible alleles(rs9265893). Similarly, in the analysis of the effect of AS on ICH, 3 SNPs were deleted (rs16894011 and rs181316459 for being palindromic, and rs9265893 for incompatible alleles). Besides, traits association analysis (see **Supplementary Table 1**) showed that 3 SNPs (rs62394289 in the SLC17A3 gene, rs9378220 in TRIM31, rs112733823 in LINC00243) were associated with other potential risk factors of stroke (high blood pressure and Rheumatoid arthritis) and therefore, were also excluded. Finally, the same 7 SNPs remained and were selected as IVs and included for analysis of stroke and its subtypes. The 7 SNPs for AS accounted for approximately 1.11% of the variation in AS. In addition, to avoid the possible effects of weak IVs, we tested the correlation strength of IVs with AS using the F statistic, and no evidence of weak IVs was identified among the chosen SNPs (all F > 10). The characteristics of the AS-associated genetic variants included in the MR study are displayed in **Table 2**.

**Table 2:** Characteristics of the AS-Associated Genetic instrumental Variants.

SNP	Nearby Gene	Ch	EA	NEA	EAF	Beta	SE	*P*	Variance, %	F
rs13033284	RP11-642D6.1	2	C	T	0.6277	-0.2214	0.0386	9.67008E-09	0.4023	671.2
rs9264277	TBC1D22B	6	C	T	0.7297	0.5198	0.0448	3.73164E-31	0.2990	498.2
rs34982906	ZFP57	6	C	T	0.05278	0.8211	0.0918	3.82296E-19	0.0714	118.7
rs9391773	NA	6	T	G	0.1285	2.6349	0.0702	1E-200	0.1220	202.9
rs79693223	TBC1D22B	6	T	C	0.04504	1.2953	0.1062	3.36279E-34	0.0533	88.7
rs76644067	CLPSL2	6	A	G	0.04745	0.7328	0.0946	9.44061E-15	0.0672	111.7
rs10807943	SLC29A4	7	C	T	0.9365	-0.5628	0.0812	4.09826E-12	0.0912	151.7

MR, Mendelian randomization; SNP, single-nucleotide polymorphism; Ch, chromosome; EA, effect allele; NEA, non-effect allele; EAF, effect allele frequency; Beta, the regression coefficient based on AS raising effect allele; SE, standardized error; Variance, proportion of variance in AS explained by each SNP. Calculated as R^2 =^ 2β2EAF(1−EAF)/2β2EAF(1−EAF) + (se(β))^2^ 2NEAF(1−EAF).

### Causal effects of AS on stroke

**Figure 2:**
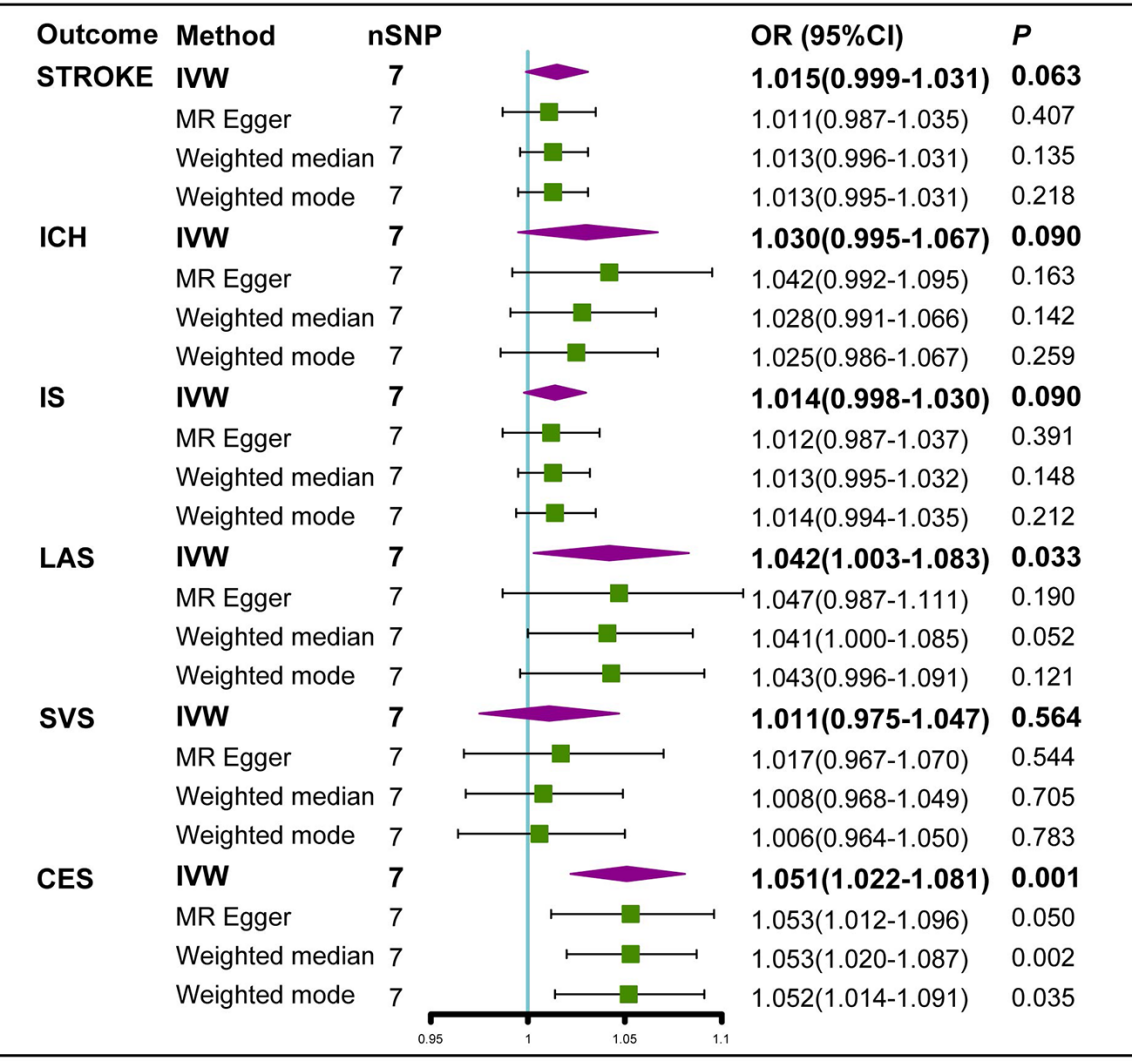
Estimated causal effects of AS on stroke using different MR methods. ICH, intracerebral hemorrhage; IS, Ischemic stroke; LAS, large artery atherosclerosis; SVS, small vessel stroke; CES cardioembolic stroke; Cl, confidence interval; IVW, inverse variance weighted; MR-Egger, Mendelian randomization-Egger; SNPs, single nucleotide polymorphisms.

Neither the IVW analysis nor the MR-Egger regression or the weighted median approaches showed genetically predicted AS to be linked with stroke, ICH, or IS (**Figure 2**). In the sensitivity analysis, there was no evidence of heterogeneity or pleiotropy among these SNPs (**Table 2**).

**Figure 3:**
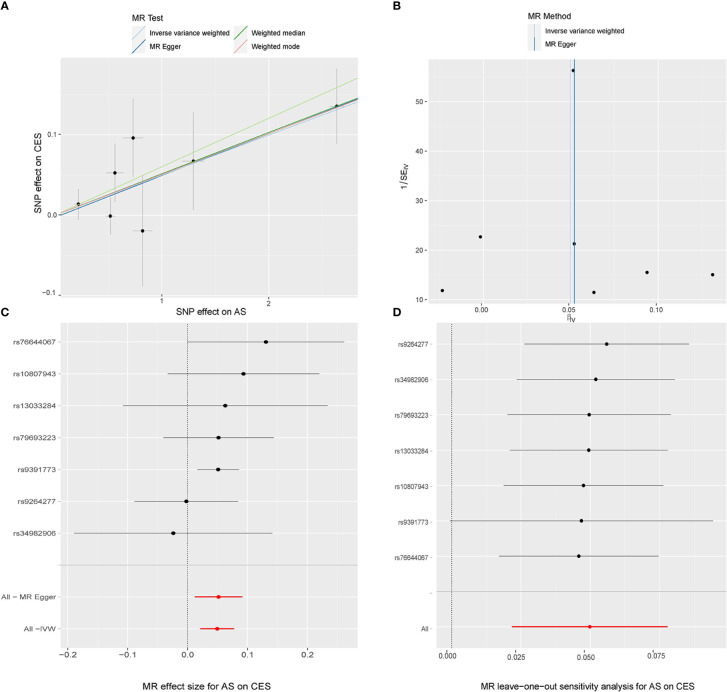
MR study of the effects of AS on CES. Scatter plot **(A)**, funnel plot **(B)**, forest plot **(C)**, and leave-one-out sensitivity analysis **(D)** of the effect of AS on CES.

**Figure 4:**
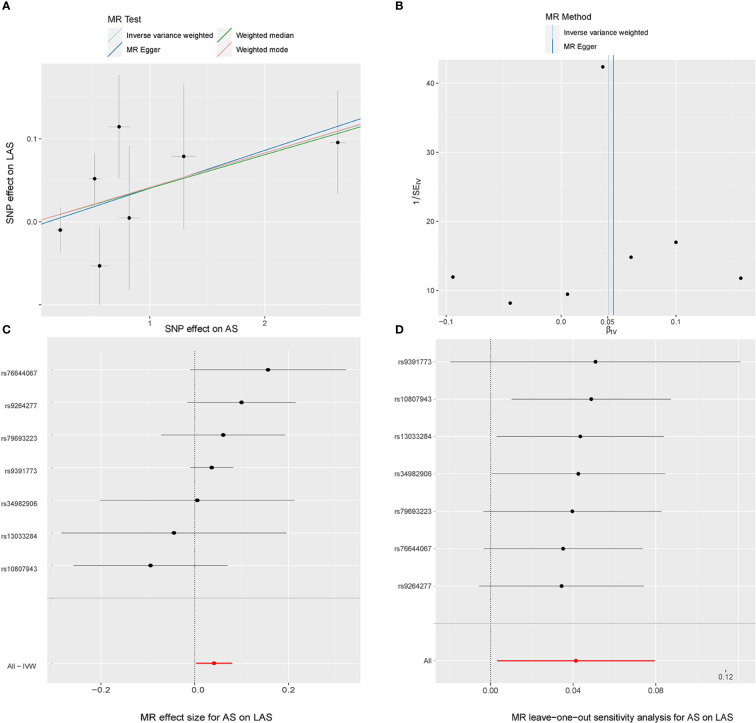
MR study of the effects of AS on LAS. Scatter plot **(A)**, funnel plot **(B)**, forest plot **(C)**, and leave-one-out sensitivity analysis **(D)** of the effect of AS on LAS.

For IS subtypes, according to the IVW technique, there was strong evidence that genetically predicted AS had a positive causal inference on CES (OR 1.051; 95% CI 1.022-1.081; P = 0.001) and suggestive evidence for the effect of genetically predicted AS on LAS (OR 1.042; 95% CI 1.003-1.082; P = 0.033), and the MR-Egger regression and weighted median methods produced comparable findings (**Figures 3A**, **4A**), despite some having lower statistical power. However, there was no evidence that genetically predicted AS had a direct influence on SVS (OR 1.010; 95% CI 0.975-1.047; P = 0.563) (**Figure 2**).

**Figure 5:**
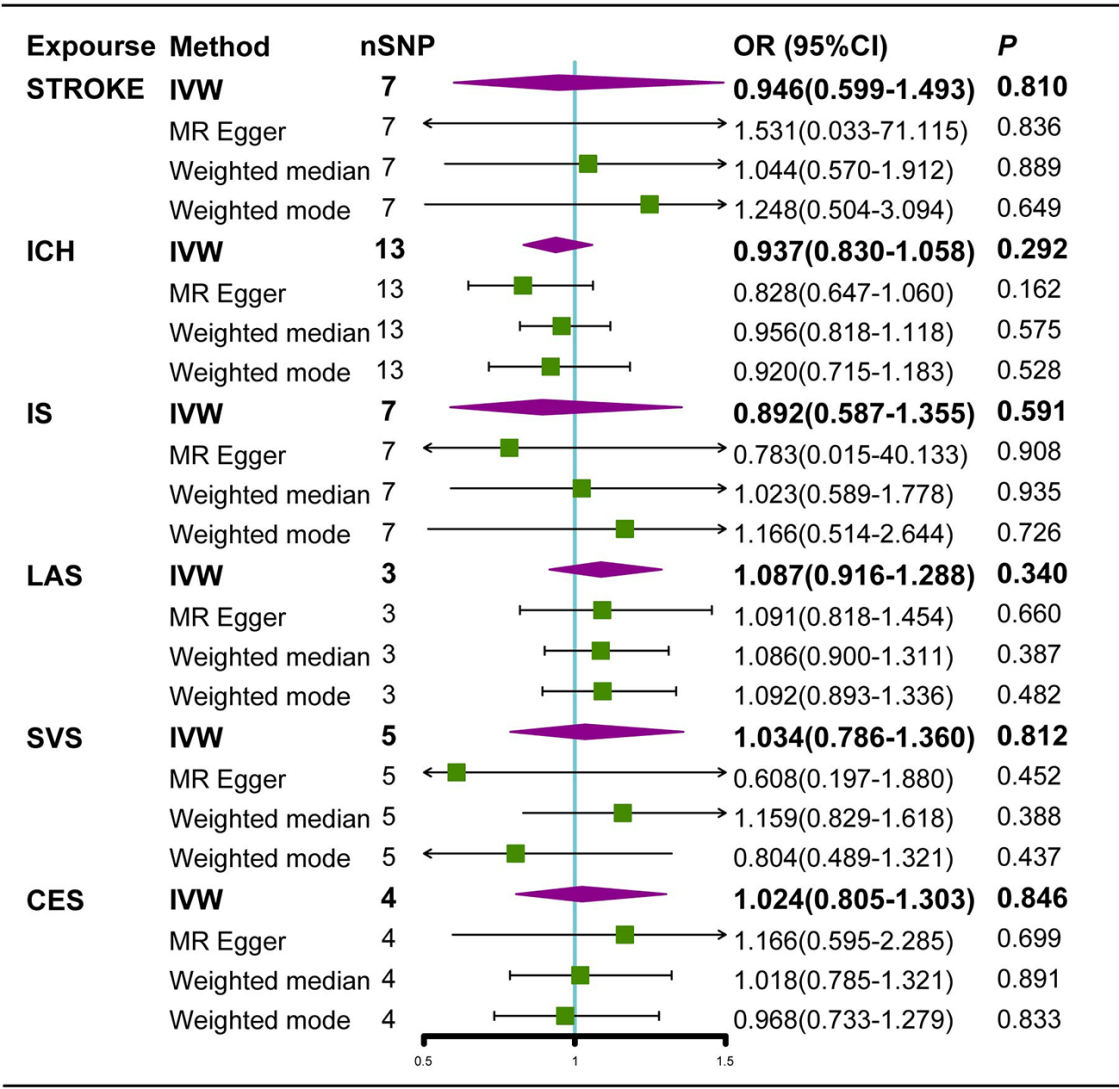
Estimated causal effects of stroke on AS using different MR methods. ICH, Intracerebral hemorrhage; IS, Ischemic stroke; LAS, large artery atherosclerosis; SVS, small vessel stroke; CES cardioembolic stroke; Cl, confidence interval; IVW, inverse variance weighted; MR-Egger, Mendelian randomization-Egger; SNPs, single nucleotide polymorphisms.

In the random-effects model, the causal effects of AS on LAS and CES were also examined and confirmed (**Table 3**). In addition, there was no evidence of horizontal pleiotropy (*P* for MR-Egger intercept 0.88 and 0.70, respectively; *P* for MR-PRESSO Global Test 0.21 and 0.67, respectively, **Table 3**) or any putative outlier SNPs in the leave-one-out analysis of AS correlations with CES or LAS (**Figures 3D**, **4D**), and Cochran’s Q statistics showed there was no heterogeneity (**Table 3**) which confirmed the reliability of the results, The funnel plots of AS with CES or LAS are shown in (**Figures 3B**, **4B**), respectively. The contributions of individual SNPs and overall estimated effects of AS with CES or LAS are shown in **Figures 3C**, **4C**, respectively. Meanwhile, our MR analyses yield sufficient power (above 90% to detect an OR of 1.30) to find the effect of genetically predicted AS on stroke and IS and moderate power to find the effect of AS on CES (74% to detect an OR of 1.30) and SVS (62% to detect an OR of 1.30), but lower power in the study of AS’s effect on LAS (53% power to detect an OR of 1.30) and ICH (25% power to detect an OR of 1.30). In addition, there was no evidence that genetic liability to stroke and its subtypes had any effect on AS (**Figure 5**), which means our results are less likely to be influenced by inverse causation. Information on IVs used in the analysis of genetically predicted stroke’s effect on AS and associated sensitivity analysis results are presented in **Supplementary Tables 2, 3**.

**Table 3:** MR Sensitivity Analyses of Genetically Predicted AS on stroke.

Outcome	No.of IVs	Heterogeneity tests		Directional horizontal pleiotropy test		IVW-MRE	
		Methods	Cochran’sQ (*P*)	MR-Egger intercept *(P*)	*P*pleiotropy*	OR (95%CI)	*P*
**STROKE**	7	MR Egger	6.31 (0.28)	4.10E-03 (0.69)	0.62	1.014 (0.999-1.031)	0.063
		IVW	6.54 (0.37)				
**ICH**	7	MR Egger	3.84 (0.57)	-1.84E-02(0.56)	0.67	1.036 (0.994-1.080)	0.090
		IVW	4.23 (0.65)				
**IS**	7	MR Egger	5.98 (0.32)	-2.20E-03 (0.84)	0.78	1.013 (0.998-1.030)	0.089
		IVW	6.04 (0.41)				
**LAS**	7	MR Egger	6.14 (0.29)	-5.41E-03 (0.10)	0.67	1.042 (1.003-1.083)	0.033
		IVW	6.19 (0.40)				
**SVS**	7	MR Egger	3.67 (0.59)	-7.27E-03(0.75)	0.9	1.010 (0.982-1.040)	0.467
		IVW	3.78 (0.70)				
**CES**	7	MR Egger	4.09 (0.53)	-2.70E-03(0.88)	0.21	1.051 (1.027-1.076)	2.68E-05
		IVW	4.11 (0.66)				

*detect by MR-PRESSO Global Test; IVs, instrumental variables; ICH, Intracerebral hemorrhage; IS, Ischemic stroke; LAS, large artery stroke; SVS, small vessel stroke; CES, cardioembolic stroke; CI, confidence interval; MR-Egger, Mendelian randomization-Egger; IVW-MRE, inverse variance weighted (multiplicative random effects).

## Discussion

Our MR analyses found evidence of a relationship between genetically predicted AS and stroke liability (for CES and LAS subtypes) indicating a potential causal link between these conditions.

The findings of the majority of case-control and observational research on the association between AS and stroke have been contentious (6, 19, 20). According to a large cohort research study by Dong Hyun Lee et al. (21), individuals with AS had a greater incidence of stroke. Throughout the follow-up period, patients with AS had a statistically significant increase (P = 0.0041) in IS risk compared to the control group (21). It should be noted, however, that they were only able to give evidence of a correlation between these two characteristics, not a causative one. In contrast to these findings, a retrospective cohort study by Sinead Brophy et al. (6) compared 1686 AS patients with 1,206,622 control participants and found no indication of a link between AS and stroke, even though patients with AS had a greater prevalence of hypertension (6). In the final analysis of this study, however, possible confounding factors such as anesthesia during surgery, body mass index, and lipoprotein cholesterol were not accounted for. In the meantime, the diagnosis of AS was reliant on medical recorders; hence, the typical examples of AS were not fully identified, which may have led to interpretation mistakes.

The studies discussed above did not investigate the influence of AS on subtypes of stroke. Given the biological rationality that AS may have different effects on different stroke subtypes, it is necessary to clarify the effects of AS on stroke subtypes with distinct etiologies. Our work extends previous studies by directly assessing the causal impact of genetically predicted AS on different subtypes of stroke based on summary data from a large-scale GWAS. We found evidence that the relationship with genetically predicted AS was specific to CES and LAS, but not SVS or all stroke. The results were overall robust to sensitivity analyses.

The basic mechanisms behind the impact of AS on stroke and its subtypes remain largely unknown. But there is some biological evidence that AS is more likely to influence the CES and LAS subtypes of strokes than the other subtypes. AS is one of several conditions that belong to a group of chronic inflammatory rheumatic disorders called spondyloarthropathies (22), which are usually marked by a state of chronic inflammation, a disturbed metabolism, oxidative stress, and endothelialdys function, which may play a role in the development of atherosclerosis and cardiovascular diseases (23–25). Atherosclerosis of the large arteries may have a causative relationship with inflammation, which is an intrinsic component of the etiology of AS. Elevated levels of inflammatory markers such as interleukin-6, C-reactive protein, and tumor necrosis factor alpha promote atherogenesis and may result in the intima and media of large and medium-sized blood arteries being thicker. (20, 26). In addition, AS is characterized by a high frequency of metabolic syndrome (27) and elevated levels of oxidative stress (28). The ensuing outcomes, including an abnormal adipokine profile (29), are reported to be causally associated with the pathophysiology of atherosclerotic cardiovascular disease (30, 31) followed by oxidative stress, like fetuin-A (32), a toll-like receptor 4 endogenous ligand by which lipids promote insulin resistance (33), contributes to an increased risk of LAS (34). CES may be the consequence of cardiomyopathy, valvular heart disease, or aortic insufficiency, which constitute extra-articular manifestations of AS (20, 35, 36). As for SVS, SVS is caused by the occlusion of small blood vessels in the brain; however, studies of the cardiovascular consequences of AS indicate that the effects may be skewed toward major arteries and cardiac tissues such as heart valves (37). To our knowledge, there are no unique clinical reports of ICH in AS. However, the underlining mechanism could be endothelial dysfunction, oxidative stress, lipid abnormalities, etc., referring to an MR study about rheumatoid arthritis and stroke (including ICH).

Our study did not detect an association between genetic liability to AS and risk of all kinds of stroke-like stroke overall, ICH, or IS, and SVS. This suggests that increased stroke risk may not only be caused by AS per se but also by non-genetic variables such as medication usage in AS patients. These negative MR results may be partially attributable to insufficient power since the power to identify an OR of 1.30 in stroke overall or IS was high, but was lower to detect the association between genetic liability to AS and SVS or ICH. Our MR investigation provided proof of a potential causal relationship between genetically predicted AS and stroke, which is most likely mediated by CES and LAS mechanisms. Further investigation is required into the mechanisms underlying the association between AS and CES or LAS. Individuals with AS may benefit from stroke prevention treatments based on CES and LAS mechanisms.

Major strengths of this work include large-scale GWAS cases and detailed analyses employing genetic instruments that were significantly linked with AS to remove reverse causality, recollection bias, and a few unidentified confounders. Multiple methods and detailed analyses increased the robustness of our conclusion. However, our study still has several limitations. First, the genetic data for exposure or outcome are GWAS summary data, which lack age- or sex-specific data, making MR analyses with age or sex stratification unavailable. Second, the proportions of stroke subtypes are well known to differ based on race (38); the most common types of stroke in European populations are CES and LAS (39), while SVS is much more common in Asian populations (40) Our findings were influenced to some extent by population susceptibility. Further research into the validity of the causal inference in other ancestry populations is needed. Third, the current investigation revealed a causal link between AS and CES, and LAS. Further study is necessary to determine if stratified-risk AS patients should get preventative therapy treatment for stroke.

## Conclusions

This research indicates that the possible causative influence of genetically predicted AS on stroke may be limited to the CES and LAS subtypes.

## Data availability statement

The original contributions presented in the study are included in the article/**Supplementary Material**. Further inquiries can be directed to the corresponding authors.

## Author contributions

JM and LZ conceived the presented idea. JM, PW, and HD developed the theory and performed the computations. WZ verified the analytical methods. JM and PW drafted the manuscript. YT and XF reviewed the manuscript. All authors discussed the results and contributed to the final manuscript.

## References

[1] Dougados M Baeten D. Spondyloarthritis. Lancet (2011) 377:2127–37. doi: 10.1016/s0140-6736(11)60071-8 21684383

[2] Bremander A Petersson IF Bergman S Englund M. Population-based estimates of common comorbidities and cardiovascular disease in ankylosing spondylitis. Arthritis Care Res (2011) 63:550–6. doi: 10.1002/acr.20408 21452267

[3] Huang Y-P Wang Y-H Pan S-L. Increased risk of ischemic heart disease in young patients with newly diagnosed ankylosing spondylitis – a population-based longitudinal follow-up study. PloS One (2013) 8:e64155. doi: 10.1371/journal.pone.0064155 23691161 PMC3655062

[4] Lee DH Choi YJ Han I Hong JB Do Han K Choi JM. Association of ischemic stroke with ankylosing spondylitis: A nationwide longitudinal cohort study. Acta Neurochir (2018) 160:949–55. doi: 10.1007/s00701-018-3499-7 29470721

[5] Kang J-H Chen Y-H Lin H-C. Comorbidity profiles among patients with ankylosing spondylitis: A nationwide population-based study. Ann Rheum Dis (2010) 69:1165–8. doi: 10.1136/ard.2009.116178 20375121

[6] Brophy S Cooksey R Atkinson M Zhou S-M Husain MJ Macey S. No increased rate of acute myocardial infarction or stroke among patients with ankylosing spondylitis–a retrospective cohort study using routine data. Semin Arthritis Rheumatism (2012) 42:140–5. doi: 10.1016/j.semarthrit.2012.02.008 22494565

[7] Liu W Ma W Liu H Li C Zhang Y Liu J. Stroke risk in arthritis: A systematic review and meta-analysis of cohort studies. PloS One (2021) 16:e0248564. doi: 10.1371/journal.pone.0248564 33725018 PMC7963101

[8] O’Donnell CJ Sabatine MS. Opportunities and challenges in mendelian randomization studies to guide trial design. JAMA Cardiol (2018) 3:967. doi: 10.1001/jamacardio.2018.2863 30326490

[9] Chen S Luo X Zhao J Liang Z Gu J. Exploring the causality between ankylosing spondylitis and atrial fibrillation: A two-sample mendelian randomization study. Front Genet (2022) 13:951893. doi: 10.3389/fgene.2022.951893 36468019 PMC9708899

[10] Adams HP Bendixen BH Kappelle LJ Biller J Love BB Gordon DL. Classification of subtype of acute ischemic stroke. definitions for use in a multicenter clinical trial. TOAST. trial of org 10172 in acute stroke treatment. Stroke (1993) 24:35–41. doi: 10.1161/01.str.24.1.35 7678184

[11] Malik R Chauhan G Traylor M Sargurupremraj M Okada Y Mishra A. Multiancestry genome-wide association study of 520,000 subjects identifies 32 loci associated with stroke and stroke subtypes. Nat Genet (2018) 50:524–37. doi: 10.1038/s41588-018-0058-3 PMC5968830 29531354

[12] Consortium T 1000. A map of human genome variation from population-scale sequencing. Nature (2010) 467:1061–73. doi: 10.1038/nature09534 PMC3042601 20981092

[13] Burgess S Thompson SG Collaboration C. Avoiding bias from weak instruments in mendelian randomization studies. Int J Epidemiol (2011) 40:755–64. doi: 10.1093/ije/dyr036 21414999

[14] Teslovich TM Musunuru K Smith AV Edmondson AC Stylianou IM Koseki M. Biological, clinical and population relevance of 95 loci for blood lipids. Nature (2010) 466:707–13. doi: 10.1038/nature09270 PMC3039276 20686565

[15] Bowden J Davey Smith G Burgess S. Mendelian randomization with invalid instruments: effect estimation and bias detection through egger regression. Int J Epidemiol (2015) 44:512–25. doi: 10.1093/ije/dyv080 PMC4469799 26050253

[16] Bowden J Davey Smith G Haycock PC Burgess S. Consistent estimation in mendelian randomization with some invalid instruments using a weighted median estimator. Genet Epidemiol (2016) 40:304–14. doi: 10.1002/gepi.21965 PMC4849733 27061298

[17] Verbanck M Chen C-Y Neale B Do R. Detection of widespread horizontal pleiotropy in causal relationships inferred from mendelian randomization between complex traits and diseases. Nat Genet (2018) 50:693–8. doi: 10.1038/s41588-018-0099-7 PMC6083837 29686387

[18] Hemani G Zheng J Elsworth B Wade KH Haberland V Baird D. The MR-base platform supports systematic causal inference across the human phenome. eLife (2018) 7:e34408. doi: 10.7554/elife.34408 29846171 PMC5976434

[19] Eriksson JK Jacobsson L Bengtsson K Askling J. Is ankylosing spondylitis a risk factor for cardiovascular disease, and how do these risks compare with those in rheumatoid arthritis? Ann Rheum Dis (2016) 76:364–70. doi: 10.1136/annrheumdis-2016-209315 27283333

[20] Lin C-W Huang Y-P Chiu Y-H Ho Y-T Pan S-L. Increased risk of ischemic stroke in young patients with ankylosing spondylitis: A population-based longitudinal follow-up study. PloS One (2014) 9:e94027. doi: 10.1371/journal.pone.0094027 24714094 PMC3979725

[21] Behari S Singh S Bhaisora KS. Ischemic stroke associated with ankylosing spondylitis: an integral part of disease spectrum, or a natural consequence of progressive infirmity? Acta Neurochir (2018) 160:959–61. doi: 10.1007/s00701-018-3501-4 29497832

[22] Sieper J Rudwaleit M Baraliakos X Brandt J Braun J Burgos-Vargas R. The assessment of SpondyloArthritis international society (ASAS) handbook: A guide to assess spondyloarthritis. Ann Rheumatic Dis (2009) 68:ii1–ii44. doi: 10.1136/ard.2008.104018 19433414

[23] Bodnár N Kerekes G Seres I Paragh G Kappelmayer J Némethné ZG. Assessment of subclinical vascular disease associated with ankylosing spondylitis. J Rheumatol (2011) 38:723–9. doi: 10.3899/jrheum.100668 21239756

[24] Peters MJL van Eijk IC Smulders YM Serne E Dijkmans BAC van der Horst-Bruinsma IE. Signs of accelerated preclinical atherosclerosis in patients with ankylosing spondylitis. J Rheumatol (2009) 37:161–6. doi: 10.3899/jrheum.090667 19955053

[25] Divecha H Sattar N Rumley A Cherry L Lowe GDO Sturrock R. Cardiovascular risk parameters in men with ankylosing spondylitis in comparison with non-inflammatory control subjects: Relevance of systemic inflammation. Clin Sci (2005) 109:171–6. doi: 10.1042/cs20040326 15801904

[26] Ayas ZÖ. Is ankylosing spondylitis a risk factor of recurrent cerebrovascular disease? Phys Med Rehabil Int (2017) 4(2):1115. doi: 10.26420/physmedrehabilint.2017.1115

[27] Malesci D Niglio A Mennillo GA Buono R Valentini G La Montagna G. High prevalence of metabolic syndrome in patients with ankylosing spondylitis. Clin Rheumatol (2006) 26:710–4. doi: 10.1007/s10067-006-0380-5 16933103

[28] Solmaz D Kozaci D Sari I Taylan A Onen F Akkoc N. Oxidative stress and related factors in patients with ankylosing spondylitis. Eur J Rheumatol (2016) 3:20–4. doi: 10.5152/eurjrheum.2015.0031 PMC5042269 27708964

[29] Gkolfinopoulou C Stratikos E Theofilatos D Kardassis D Voulgari PV Drosos AA. Impaired antiatherogenic functions of high-density lipoprotein in patients with ankylosing spondylitis. J Rheumatol (2015) 42:1652–60. doi: 10.3899/jrheum.141532 26233507

[30] Ference BA Ginsberg HN Graham I Ray KK Packard CJ Bruckert E. Low-density lipoproteins cause atherosclerotic cardiovascular disease. 1. Evidence from genetic, epidemiologic, and clinical studies. A consensus statement from the European Atherosclerosis Society Consensus Panel. Eur Heart J. (2017) 38(32):2459–72. doi: 10.1093/eurheartj/ehx144 PMC5837225 28444290

[31] Yuan S Tang B Zheng J Larsson SC. Circulating lipoprotein lipids, apolipoproteins and ischemic stroke. Ann Neurol (2020) 88:1229–36. doi: 10.1002/ana.25916 PMC7756401 32981134

[32] Sari I Kebapcilar L Taylan A Bilgir O Kozaci DL Yildiz Y. Fetuin-a and interleukin-18 levels in ankylosing spondylitis. Int J Rheumatic Dis (2010) 13:75–81. doi: 10.1111/j.1756-185x.2009.01448.x 20374388

[33] Pal D Dasgupta S Kundu R Maitra S Das G Mukhopadhyay S. Fetuin-a acts as an endogenous ligand of TLR4 to promote lipid-induced insulin resistance. Nat Med (2012) 18:1279–85. doi: 10.1038/nm.2851 22842477

[34] Rittig K Thamer C Haupt A Machann J Peter A Balletshofer B. High plasma fetuin-a is associated with increased carotid intima-media thickness in a middle-aged population. Atherosclerosis (2009) 207:341–2. doi: 10.1016/j.atherosclerosis.2009.05.018 19615685

[35] Brunner F Kunz A Weber U Kissling R. Ankylosing spondylitis and heart abnormalities: Do cardiac conduction disorders, valve regurgitation and diastolic dysfunction occur more often in male patients with diagnosed ankylosing spondylitis for over 15 years than in the normal population? Clin Rheumatol (2005) 25:24–9. doi: 10.1007/s10067-005-1117-6 16247583

[36] Ozkan Y. Cardiac involvement in ankylosing spondylitis. J Clin Med Res (2016) 8:427–30. doi: 10.14740/jocmr2488w PMC4852774 27222669

[37] Heeneman S Daemen MJ. Cardiovascular risks in spondyloarthritides. Curr Opin Rheumatol (2007) 19:358–62. doi: 10.1097/bor.0b013e328133f58e 17551366

[38] White H Boden-Albala B Wang C Elkind MSV Rundek T Wright CB. Ischemic stroke subtype incidence among whites, blacks, and hispanics. Circulation (2005) 111:1327–31. doi: 10.1161/01.cir.0000157736.19739.d0 15769776

[39] Stewart JA Dundas R Howard RS Rudd AG Wolfe CDA. Ethnic differences in incidence of stroke: Prospective study with stroke register. BMJ (1999) 318:967–71. doi: 10.1136/bmj.318.7189.967 PMC27822 10195965

[40] Banerjee S Biram R Chataway J Ames D. South Asian strokes: Lessons from the St mary’s stroke database. QJM (2009) 103:17–21. doi: 10.1093/qjmed/hcp148 19843602

