# Supplementary material for: Impact of ankylosing spondylitis on stroke limited to specific subtypes: Evidence from Mendelian randomization study
Source: Front Immunol. 2023 Jan 19;13:1095622. doi: 10.3389/fimmu.2022.1095622 (PMC9893629; doi:10.3389/fimmu.2022.1095622)
Supplement: Supplementary file 1 [file DataSheet_1.docx]

Impact of ankylosing spondylitis on stroke limited to specific subtypes: evidence from Mendelian Randomization Study

The Supplementary Materials include:

1) Supplementary Method

2) Supplementary Table 1-3

**Supplementary Method**

**Genetic Instrument Selection of stroke and its subtypes**

Similar to the selection of instrumental variables (IVs) for AS in the genetic prediction of AS and stroke study. The selection of IVs for stroke and its subtypes involves six steps. Different is that there are no IVs for some stroke subtypes in the first phase according to the genome-wide significance criterion (*P*＜5E–8). Thus, the following criteria were established: For stroke, LAS, and CES, the threshold for selecting suitable IVs was set at *P*＜5E–8. For SVS, the threshold for selecting eligible IVs was established at *P*＜5E–7. For ICH, the threshold for selecting suitable IVs was set at *P*＜1E–5. The remaining five steps are identical to those detailed in the manuscript. Analysis of trait associations found that none of these IVs were associated with other possible AS risk factors (not shown).

**Supplementary Table 1.** The association between instrumental SNPs of AS with traits by LD trait.

| SNP | Population | Associated phenotypes in PhenoScanner_V2_ |
| --- | --- | --- |
| rs13033284^#^ | European | NA |
| rs9264277^#^ | European | Monocyte count, Monocyte percentage of, white cells, White blood cell count, Granulocyte percentage of, myeloid white cells, Lymphocyte count, Myeloid white cell count, Mean corpuscular hemoglobin, Mean corpuscular volume. |
| rs34982906^#^ | European | NA |
| rs9391773^#^ | European | NA |
| rs79693223^#^ | European | NA |
| rs76644067^#^ | European | NA |
| rs10807943^#^ | European | NA |
| rs62394289* | European | Self-reported hypertension, Vascular or heart problems diagnosed by doctor: high blood pressure, Diastolic blood pressure, Mean corpuscular hemoglobin, Mean corpuscular volume, Red cell distribution width, Mean corpuscular hemoglobin concentration, Hemoglobin concentration, Treatment with blood pressure medication, Vascular or heart problems diagnosed by doctor: none of the above, Reticulocyte fraction of red cells, Red blood cell count |
| rs9378220^*^ | European | Rheumatoid arthritis, Immature fraction of reticulocytes, High light scatter reticulocyte count, High light scatter percentage of red cells, Height, Reticulocyte count, Mean corpuscular hemoglobin, Self-reported malabsorption or, coeliac disease, Red blood cell count |
| rs112733823^*^ | European | Rheumatoid arthritis, Mean platelet volume |

SNP, single nucleotide polymorphism. **^#^** SNP retained as IVs of AS. ^*^SNP deleted for associated with other potential risk factors of stroke (high blood pressure and Rheumatoid arthritis)

**Supplementary Table 2.** Sensitivity analysis on the association between genetically predicted stroke and risk of AS.

| Exposure | No.of  IVs | Heterogeneity tests | | Directional horizontal pleiotropy test | | IVW-MRE | |
| --- | --- | --- | --- | --- | --- | --- | --- |
|  |  | **Methods** | **Cochran'sQ (*P*)** | **MR-Egger intercept *(P*)** | ***P*pleiotropy*** | **OR（95%CI）** | ***P*** |
| STROKE | 7 | MR Egger | 5.52 (0.36) | 4.10E-03 (0.81) | 0.496 | 0.946(0.608-1.470) | 0.803 |
|  |  | IVW | 5.58 (0.47) |  |  |  |  |
| ICH | 13 | MR Egger | 10.37(0.50) | 3.71E-02 (0.28) | 0.51 | 0.937(0.831-1.056) | 0.284 |
|  |  | IVW | 11.64 (0.48) |  |  |  |  |
| IS | 7 | MR Egger | 6.00 (0.31) | 9.61E-03(0.95) | 0.45 | 0.892(0.587-1.355) | 0.591 |
|  |  | IVW | 6.01 (0.42) |  |  |  |  |
| LAS | 3 | MR Egger | 0.39 (0.53) | -1.71E-03(0.98) | NONE# | 1.087(1.008-1.172) | 0.031 |
|  |  | IVW | 0.39 (0.82) |  |  |  |  |
| SVS | 5 | MR Egger | 2.17 (0.54) | 9.54E-02(0.41) | 0.53 | 1.034(0.813-1.314) | 0.786 |
|  |  | IVW | 3.07 (0.55) |  |  |  |  |
| CES | 4 | MR Egger | 3.14 (0.21) | -2.95E-02(0.72) | 0.47 | 1.024(0.805-1.303) | 0.846 |
|  |  | IVW | 3.41 (0.33) |  |  | 0.946(0.608-1.470) | 0.803 |

Note: * detect by MR-PRESSO Global Test; IVs: instrumental variables; ICH, Intracerebral hemorrhage; IS, Ischemic stroke; LAS, large artery stroke; SVS, small vessel stroke; CES, cardioembolic stroke; CI, confidence interval; MR-Egger, Mendelian randomization-Egger; IVW-MRE, inverse variance weighted (multiplicative random effects). **^#^** Not enough IVs to conduct MR-PRESSO Global Test.

**Supplementary Table 3.** Characteristics of the stroke-Associated Genetic instrumental Variants.

| SNP | Nearby Gene | Ch | EA | NEA | EAF | Beta | SE | *P* | Variance, % | F |
| --- | --- | --- | --- | --- | --- | --- | --- | --- | --- | --- |
| Instrumental variables of stroke | | | | | | | | | | |
| rs1052053 | NA | 1 | G | A | 0.3811 | -0.0675 | 0.0096 | 2.25E-12 | 0.023715 | 10850.6 |
| rs11242678 | RP11-157J24.2 | 6 | T | C | 0.2551 | 0.0643 | 0.0105 | 8.71E-10 | 0.019901 | 9070.3 |
| rs2107595 | HDAC9 | 7 | A | G | 0.1671 | 0.0803 | 0.0121 | 3.59E-11 | 0.01506 | 6830.1 |
| rs1537375 | CDKN2B-AS1 | 9 | C | T | 0.5021 | 0.0519 | 0.0091 | 1.24E-08 | 0.026322 | 12075.8 |
| rs475937 | WTAPP1 | 11 | C | A | 0.8682 | -0.0757 | 0.0137 | 2.92E-08 | 0.011787 | 5327.9 |
| rs10774624 | RP3-473L9.4 | 12 | A | G | 0.5285 | -0.0654 | 0.0094 | 4.04E-12 | 0.02471 | 11317.3 |
| rs4942561 | LRCH1 | 13 | T | G | 0.7581 | 0.064 | 0.0107 | 2.05E-09 | 0.019178 | 8734.3 |
| Instrumental variables of ICH | | | | | | | | | | |
| rs77306002 | RP5-936J12.1 | 1 | C | T | 0.00767 | 1.0065 | 0.2271 | 9.37E-06 | 9.56E-05 | 19.4 |
| rs1214072 | RN7SKP102 | 2 | C | A | 0.5174 | -0.1575 | 0.0352 | 7.76E-06 | 0.003963 | 807.1 |
| rs34142414 | LINC00882 | 3 | C | T | 0.1365 | 0.2293 | 0.0516 | 8.83E-06 | 0.001848 | 375.6 |
| rs11716071 | LINC00882 | 3 | G | T | 0.4337 | -0.1637 | 0.0358 | 4.96E-06 | 0.003832 | 780.2 |
| rs11733974 | RP11-119H12.3 | 4 | C | T | 0.02921 | 0.4855 | 0.109 | 8.45E-06 | 0.000415 | 84.2 |
| rs73079569 | CDCA7L | 7 | A | G | 0.02354 | 0.5397 | 0.1217 | 9.20E-06 | 0.000333 | 67.5 |
| rs67530445 | TNFRSF19 | 13 | T | C | 0.1857 | 0.2109 | 0.0456 | 3.68E-06 | 0.002365 | 480.9 |
| rs74551590 | RP11-76E12.1 | 14 | C | T | 0.06911 | 0.3216 | 0.0716 | 7.07E-06 | 0.000961 | 195.1 |
| rs77456902 | AGBL1 | 15 | C | T | 0.02442 | 0.5352 | 0.1204 | 8.78E-06 | 0.00034 | 69.0 |
| rs80310505 | VPS13C | 15 | C | T | 0.01007 | 1.0367 | 0.1948 | 1.03E-07 | 0.00013 | 26.4 |
| rs9932700 | LA16c-444G7.1 | 16 | C | T | 0.6055 | -0.1636 | 0.0362 | 6.09E-06 | 0.003748 | 763.1 |
| rs62072393 | rs62072393 | 17 | T | C | 0.1038 | 0.2675 | 0.0588 | 5.40E-06 | 0.001424 | 289.2 |
| rs2319176 | NA | 22 | C | T | 0.07776 | 0.3039 | 0.0667 | 5.26E-06 | 0.001107 | 224.8 |
| Instrumental variables of IS | | | | | | | | | | |
| rs2758612 | PMF1-BGLAP | 1 | C | T | 0.3547 | -0.0653 | 0.0111 | 3.68E-09 | 0.018099 | 8116.2 |
| rs2066864 | FGG | 4 | A | G | 0.2452 | 0.0634 | 0.0115 | 3.51E-08 | 0.016882 | 7561.4 |
| rs11242678 | RP11-157J24.2 | 6 | T | C | 0.255 | 0.0723 | 0.0114 | 2.70E-10 | 0.017175 | 7694.6 |
| rs2107595 | HDAC9 | 7 | A | G | 0.1673 | 0.0882 | 0.0132 | 2.33E-11 | 0.012866 | 5739.2 |
| rs473238 | WTAPP1 | 11 | C | T | 0.8674 | -0.0831 | 0.0147 | 1.65E-08 | 0.0104 | 4627.7 |
| rs3184504 | SH2B3 | 12 | C | T | 0.5278 | -0.0779 | 0.0101 | 1.23E-14 | 0.021778 | 9802.9 |
| rs4942561 | LRCH1 | 13 | T | G | 0.759 | 0.0655 | 0.0116 | 1.77E-08 | 0.016597 | 7431.6 |
| Instrumental variables of LAS | | | | | | | | | | |
| rs7610618 | SIAH2 | 3 | T | C | 0.013 | 0.8449 | 0.149 | 1.44E-08 | 0.00011 | 45.0 |
| rs2107595 | HDAC9 | 7 | A | G | 0.1677 | 0.2358 | 0.0319 | 1.44E-13 | 0.002388 | 982.7 |
| rs10820405 | LINC01492 | 9 | A | G | 0.1847 | -0.1812 | 0.0331 | 4.51E-08 | 0.002219 | 912.7 |
| Instrumental variables of SVS | | | | | | | | | | |
| rs149163995 | CARF | 2 | T | C | 0.1256 | -0.1945 | 0.0368 | 1.22E-07 | 0.003715 | 738.4 |
| rs7766042 | RP11-157J24.2 | 6 | C | T | 0.1016 | 0.2129 | 0.0397 | 7.97E-08 | 0.003193 | 634.5 |
| rs76576182 | DLX5 | 7 | G | A | 0.0212 | 0.4436 | 0.0844 | 1.46E-07 | 0.000708 | 140.4 |
| rs35818742 | ADRB1 | 10 | C | T | 0.1344 | 0.1674 | 0.0329 | 3.65E-07 | 0.004643 | 923.9 |
| rs12445022 | RP11-482M8.1 | 16 | A | G | 0.3367 | 0.1301 | 0.0244 | 9.26E-08 | 0.00841 | 1679.6 |
| Instrumental variables of CES | | | | | | | | | | |
| rs146390073 | RGS7 | 1 | T | C | 0.0215 | 0.6688 | 0.1195 | 2.20E-08 | 0.000169 | 70.0 |
| rs2466455 | RP11-119H12.3 | 4 | T | C | 0.7826 | -0.2992 | 0.0222 | 2.75E-41 | 0.004885 | 2029.0 |
| rs6838973 | RP11-119H12.3 | 4 | T | C | 0.4341 | -0.1079 | 0.0196 | 3.58E-08 | 0.006259 | 2603.1 |
| rs12932445 | ZFHX3 | 16 | C | T | 0.1805 | 0.1758 | 0.0245 | 6.88E-13 | 0.004015 | 1666.0 |

MR, Mendelian randomization; SNP, single-nucleotide polymorphism; Ch, chromosome; EA, effect allele; NEA, non-effect allele; EAF, effect allele frequency; Beta, the regression coefficient based on AS raising effect allele; SE, standardized error; Variance, proportion of variance in AS explained by each SNP. Calculated as R^2^=2β2EAF(1−EAF)/2β2EAF(1−EAF) + (se(β))^2^2NEAF(1−EAF). IS, Ischemic stroke; ICH, Intracerebral haemmorrhage; LAS, large artery atherosclerosis; SVS, small vessel stroke; CES cardioembolic stroke.
